# Supplementary material for: Sequential fear generalization and network connectivity in trauma exposed humans with and without psychopathology
Source: Commun Biol. 2022 Nov 21;5:1275. doi: 10.1038/s42003-022-04228-5 (PMC9681725; doi:10.1038/s42003-022-04228-5)
Supplement: Supplementary file 2 — Supplementary Material [file 42003_2022_4228_MOESM2_ESM.pdf]

Supplementary Information for

Sequential Fear Generalization and Network Connectivity in Trauma Exposed Humans With and Without Psychopathology

This PDF file includes:

Supplementary text
Figures S1 to S6
Tables S1 to S2

Supplementary Information Text

Methods

Participants

To ensure substantial range of psychopathology in the TE sample, and consistent with a large body of work suggesting dose-response relationships between trauma exposure severity and psychopathology (29), we methodically characterized type, severity, number and timing of trauma exposures, and included both childhood and adulthood trauma exposure. Index trauma exposure met DSM-5 Criterion A of a traumatic event.

An independent MD or PhD/PsyD clinical evaluator administered the Structured Clinical Interview for DSM-5 Disorders (SCID-5)(30) and the Clinician-Administered PTSD Scale-5 (CAPS-5) (31, 32). The CAPS-5 is a 30-item instrument containing 0-4 Likert-style items of frequency and intensity for PTSD symptoms. It has good reliability and validity (33). Depressive symptoms were assessed using the Hamilton Depression Scale (HAM-D, 17 item) (34), a reliable clinician-rated measure of depressive severity. Social anxiety symptoms were assessed using the Liebowitz Social Anxiety Scale (LSAS), a well-validated 24-item clinician-rated scale for Social Anxiety Disorder (SAD) severity, widely used in studies of SAD (35). Panic disorder symptoms were assessed using the Panic Disorder Severity Scale (PDSS)(36), a self-report measure for panic severity. Self-rated instruments also included the Generalized Anxiety Disorder assessment (GAD-7) (37), and the SF-36 (38), a 36 item measure of generic health status, designed for use in clinical practice and research (39). Out of the 62 TE participants, 26 had PTSD, 11 had MDD, 5 had persistent depressive disorder (PDD), 3 had PD and 1 had SAD (See table 1).

Trauma-exposed participants were excluded due to: 1) Prior or current diagnosis of schizophrenia, psychotic disorder, bipolar disorder, dementia; 2) HAM-D score> 25 reflecting significant depression and/or depression related impairment that warranted pharmacotherapy or combined medication and psychotherapy; 3) Individuals at risk for suicide; 4) History of substance/alcohol dependence within the past six months, or abuse within past two months; 5) Any psychotropic medications including antipsychotic, antidepressant, mood stabilizer, or stimulant medications in the last four weeks prior to

the study (6 weeks for fluoxetine); 6) Pregnancy; 7) Medical illness that could interfere with assessment of response or biological measures (fMRI); 8) Paramagnetic metallic implants or devices contraindicating magnetic resonance imaging or any other non-removable paramagnetic metal in the body; and 9) Significant claustrophobia that would preclude ability to remain calm within the MRI scanner.

Healthy control participants were excluded due to: 1) Any current or past psychiatric diagnosis; 2) A history of trauma exposure that fulfills DSM-5 PTSD criteria A; 3) HAM-D score> 7; 4) Lifetime history of substance/alcohol dependence or abuse history; and point 6) to 9) as above.

fMRI Task

Five checkerboard-textured counterphase-flickering (10 Hz) rings of parametrically increasing size and one 'V-shaped' stimulus of the same counterphase-flickering type served as conditioned stimulus-type (CS+ and CS-) and generalization stimulus-type (GSs). The dimensions and size increments for employed rings are described in Figure 8. The current paradigm included one CS+ and the following two CS-: (i) either the largest or smallest ring referred to as the oCS- and (ii) a 'v–shaped' stimulus referred to as the vCS-. While all participants were conditioned with the same vCS-, the oCS- was the largest ring for 50% of participants and the smallest ring for the remaining half. Participants for whom the oCS- was the largest ring were conditioned with the smallest ring as CS+, and vice versa. The three intermediately sized rings served as GSs (i.e., GS1, GS2 and GS3) and formed a continuum-of-size between the CS+ and oCS- with GS3, GS2 and GS1 demarcating the GS with most to least similarity to the CS+, regardless of CS+ size. The inclusion of the vCS- allows for an assessment of brain responses to the CS+ (vs vCS-) that are independent of generalization effects to all ringed stimulus-type. Such an assessment is important because brain activations to the CS+ was used as functional regions of interest in which to test gradients of threat generalization and should thus be orthogonal to the generalization process. The CS+ vs vCS- contrast provides such an index of conditioning that is independent of generalization effects.

Procedure

Participants were not instructed of the CS/US contingency but were told they might learn to predict the shock if they attend to the presented stimulus-type. Shock electrodes were then attached, and the shock workup procedure was completed. Next, participants practiced using the button box to respond to the red crosshairs, which appeared both at the center of CSs and GSs and during the ITI periods. Participants were then placed in the scanner. Structural scans were acquired followed by pre-acquisition, acquisition, and generalization test. Participants rated their anxiety to CS+, oCS- and vCS- after pre-acquisition, acquisition, and generalization between scans.

All CSs and GSs were presented for 4 s on a rear-projection viewing screen mounted at the foot of the scanner with a viewing distance of 6.71 feet (204.47 cm). Inter-trial-intervals for CSs and GSs were either 2.4 or 4.8 s, during which participants focused their gaze on crosshairs in the center of the screen. The unconditioned stimulus (US) was a 100 ms electric shock (3–5mA) delivered to the participant's right ankle. Prior to the start of the experiment, a sample shock procedure was performed during which participants received between one and three sample shocks and a level of shock rated by participants as being 'highly uncomfortable but not painful' was established. Shock intensity varied by participant and had an average intensity of 4.6mA (s.d.=0.80).

Image Preprocessing
All fMRI images were preprocessed using MATLAB version R2018a (The MathWorks, Inc., Natick, Massachusetts) and statistical parametric mapping software (SPM12; Welcome Trust Centre for Neuroimaging, UCL, London, United Kingdom). (1) Functional images were spatially realigned to the first image in the time series using a six-parameter rigid body transformation; (2) then slice-time correction was performed; (3) outlier detection was carried out using artifact removal tools (ART). The principal component-based noise-correction method, "CompCor," implemented in this toolbox, was used for additional control of physiological noise and head motion effects. Outlier volumes in each participant were identified as having large spiking artifacts (i.e., volumes >3 standard deviations from the mean image intensity), or large motion (i.e., 0.5 mm for scan-to-scan head-motion composite changes in the x, y, or z direction); (4) each functional image was then spatially normalized to the standard T1 template included in SPM12; functional images were then resliced to 2 × 2 × 2 mm voxels, according to the resulting spatial realignment and normalization parameters; (5) anatomical images were segmented into grey matter, white matter, and cerebrospinal fluid (CSF) regions; (6) functional scans were smoothed with an 8mm full-width-at-half-maximum (FWHM) Gaussian kernel; (7) covariates corresponding to head motion (6 realignment parameters and their derivatives), outliers (one covariate per outlier consisting of 0s everywhere and a 1 for the outlier time point), and the BOLD time series from the participant-specific white matter and CSF masks were used in the GLM and connectivity analysis as predictors of no interest, and were removed from the BOLD functional time series using linear regression.

From the original 114 sample, four participants were excluded due to being late for scan and not able to finish the SG task (two participants), or task failure of delivering shock (two participants). Twenty-two additional participants (15 TE and 7 HC) were excluded with greater risk rating to vCS-, compared to CS+ during acquisition phase in the fMRI task, or greater rating to vCS-, compared to red cross in post questionnaires. No participant was excluded from further analysis because of movement exceeding ±1 mm. Consequently, the final neural imaging analysis included 88 participants: 62 TE and 26 HC. Sum of root mean square (RMS) of 6 relative head motion parameters (movement from this time point to the next one) was calculated for each participant in all groups (TEPG, TEHC, and HC). No significant difference in head motion was found between each pair of groups ($p>0.5$). Linear regression was performed to study the linear relationship between the dependent variable (sum of head motion) and independent variable (groups). The regression analysis results showed that the total head motion could not predict groups ($p>0.05$).

fMRI Network Analysis

Two data reduction steps were performed using principal component analysis (PCA). First, participant-specific PCA was performed to reduce the dimensionality of each participant's functional data (31). Second, participant data were concatenated into one group and PCA was performed again prior to performing ICA. Independent components, or networks, were calculated using the Infomax algorithm (32). Number of independent components were estimated from the fMRI data by using the minimum description length (MDL) criteria, yielding 30 components. The infomax algorithm was repeated fifty times with randomly initialized decomposition matrices and the same convergence threshold using the ICASSO approach to assess the reliability of the generated components (33). For each IC the "centroid" (i.e., the most stable result) was determined following the

agglomerative hierarchical clustering with average-linkage criterion, and its consistency was calculated with a cluster quality index (*Iq*) ranging from 0 to 1 (33). Single-participant component time courses were then back reconstructed using the GICA-3 back-reconstruction approach.

A systematic approach was used to identify non-artifactual ICs, or intrinsic connectivity networks (ICNs) (34). First, the *Iq* index from ICASSO was assessed as the criterion to validate the IC decomposition stability. Components with an *Iq* value less than 0.7 from 50 ICASSO repetitions were excluded. Second, visual evaluation of IC spatial patterns (e.g., ringing) as well as frequency inspection of IC time course spectra (e.g., time courses vastly dominated by low-frequency fluctuations) allowed additional components related to artifacts to be excluded from analysis. All the ICs that involved the majority of activation falling outside the cerebral cortex, for instance in the spinal cord, eyes, borders of the skull, ventricles, etc., were considered noise components and excluded from further analyses. After careful visual inspection of the spatial-temporal characteristics of each IC, 6 components were categorized as noise components, leaving 24 components for further analyses. Identification of the remaining components was accomplished by performing spatial correlation with publicly available GIFT network templates (35). Each IC was correlated with the given templates and best network template was selected based on the maximum correlation values. To define significant brain regions in each non-artifactual component, the spatial map of each component was *z*-scored to facilitate between-subjects' comparisons, and the averaged maps of *z*-scores were entered into the second-level random effects analysis in SPM12. The significance threshold was set at family-wise error (FWE)-corrected threshold of $p < 0.05$ for multiple comparisons of voxel-wise whole-brain analysis.

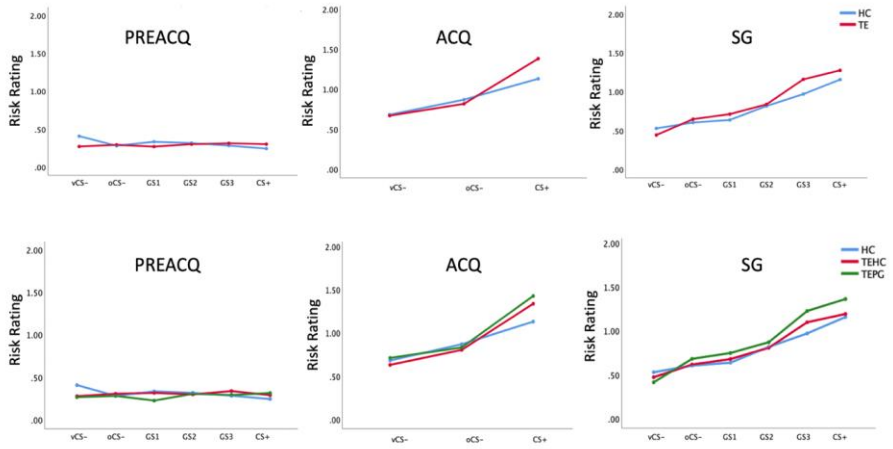

Figure S1: Mean behavioral rRisk ratings for pre-ACQ phase, ACQ phase and SG phase for two group (top: HCs, TEs) and three groups (bottom: HCs, TEHCs, TEPGs). Results suggested all groups learned the CS contingencies.

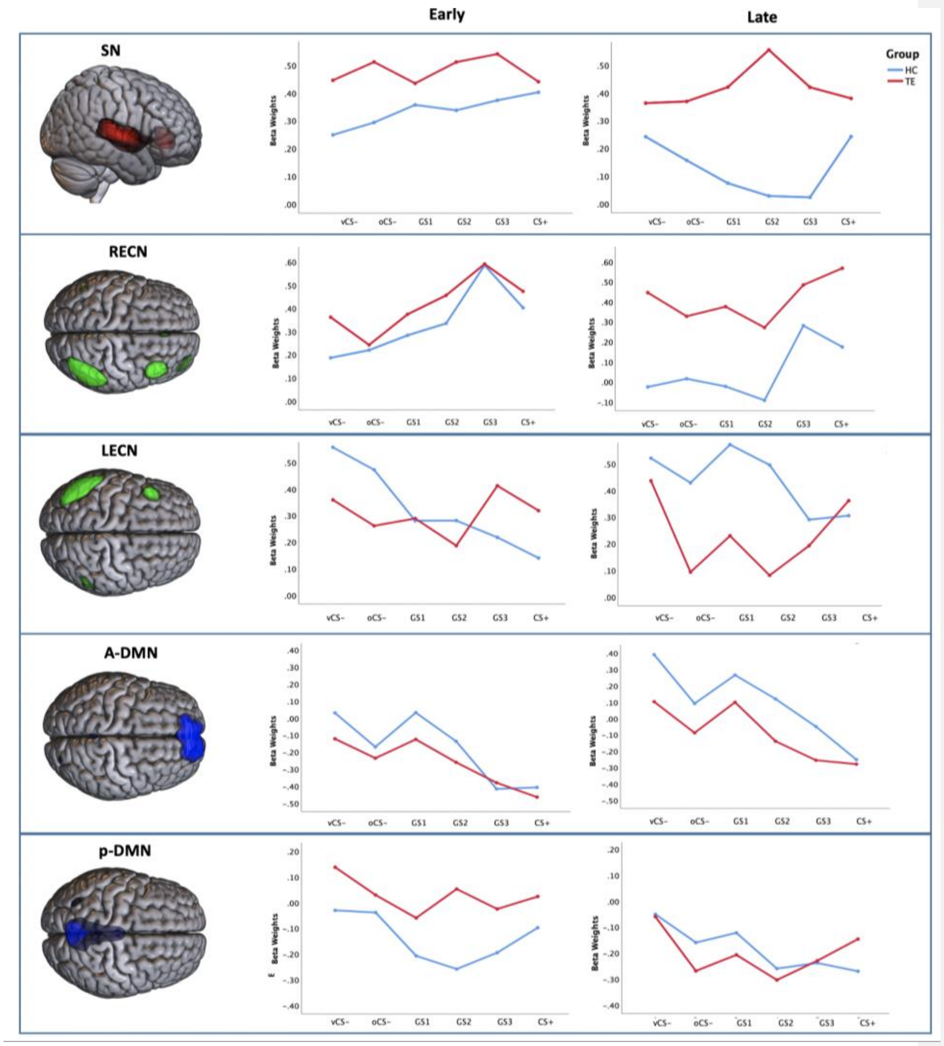

Figure S2: Neural responses to conditioned and generalization stimuli (vCS-, oCS-, GS1, GS2, GS3, CS+) across ICNs by two stages across HC and TE groups.
Abbreviation: CS+: conditioned stimuli, danger cue; oCS-: conditioned stimuli, o shape safe cue; vCS-: conditioned stimuli, v shape safe cue; GS: generalization stimuli; SN: salience network; RECN: right executive control network; LECN: left executive control network; A-DMN: anterior default mode network; p-DMN: posterior default mode network; HC: non-trauma-exposed healthy controls ; TEHC: trauma-exposed healthy controls; TEPG: trauma-exposed psychopathology group.

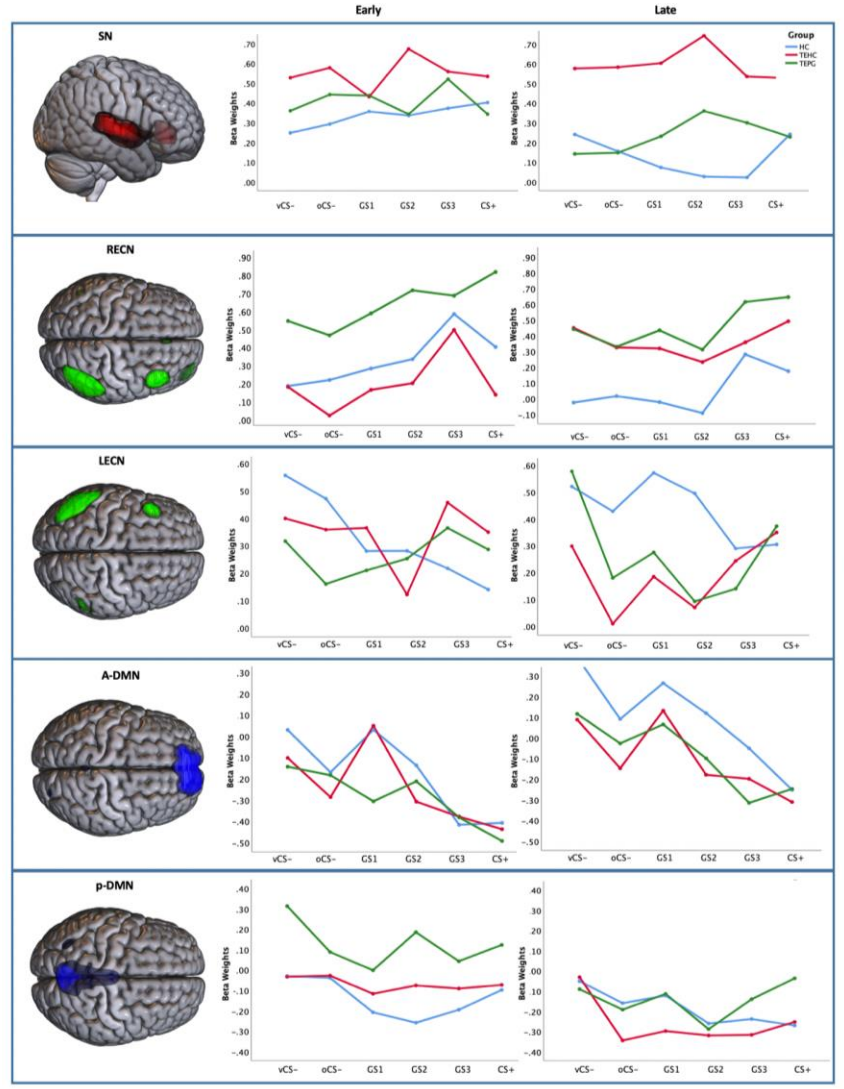

Figure S3: Neural responses to conditioned and generalization stimuli (vCS-, oCS-, GS1, GS2, GS3, CS+) across ICNs by two stages across HC and TEHC and TEPG groups.

Abbreviation: CS+: conditioned stimuli, danger cue; oCS-: conditioned stimuli, o shape safe cue; vCS-: conditioned stimuli, v shape safe cue; GS: generalization stimuli; SN: salience network; RECN: right executive control network; LECN: left executive control network; A-DMN: anterior default mode network; p-DMN: posterior default mode network; HC: non-trauma-exposed healthy

Formatted: Centered

controls ; TEHC: trauma-exposed healthy controls; TEPG: trauma-exposed psychopathology group.

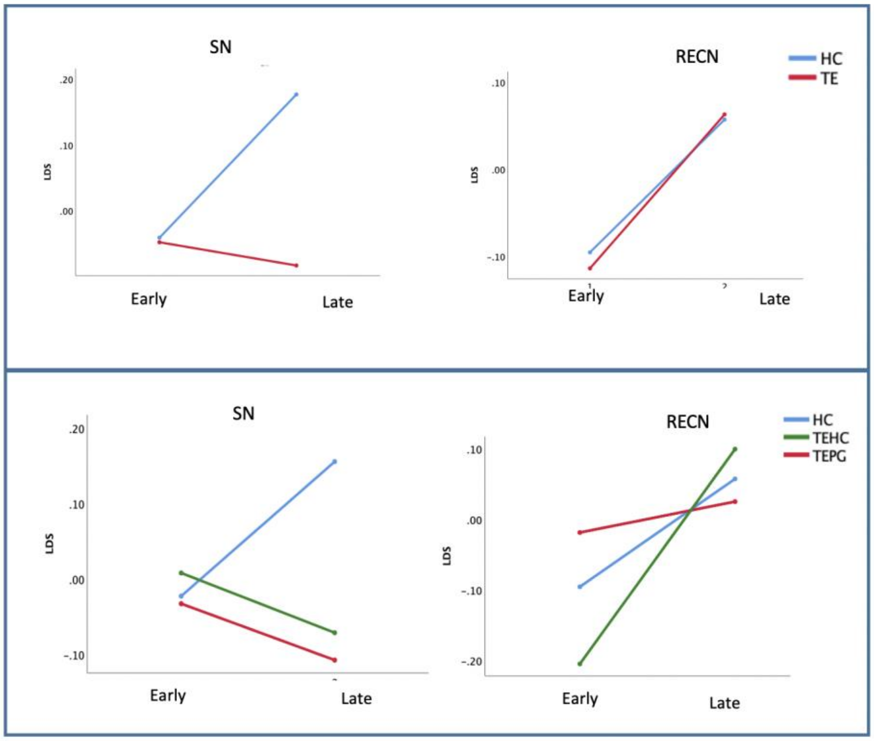

Figure S4: LDS across two stages in different groups for SN and RECN.
We assessed the changes in steepness of the generalization gradients across early and late stages (i.e., changes/ delta of generalization magnitudes: EG-LG) measured by linear deviation scores (LDS). SN: Salience network, RECN: right executive control network.~~LDS across two stages in different groups for SN and RECN~~

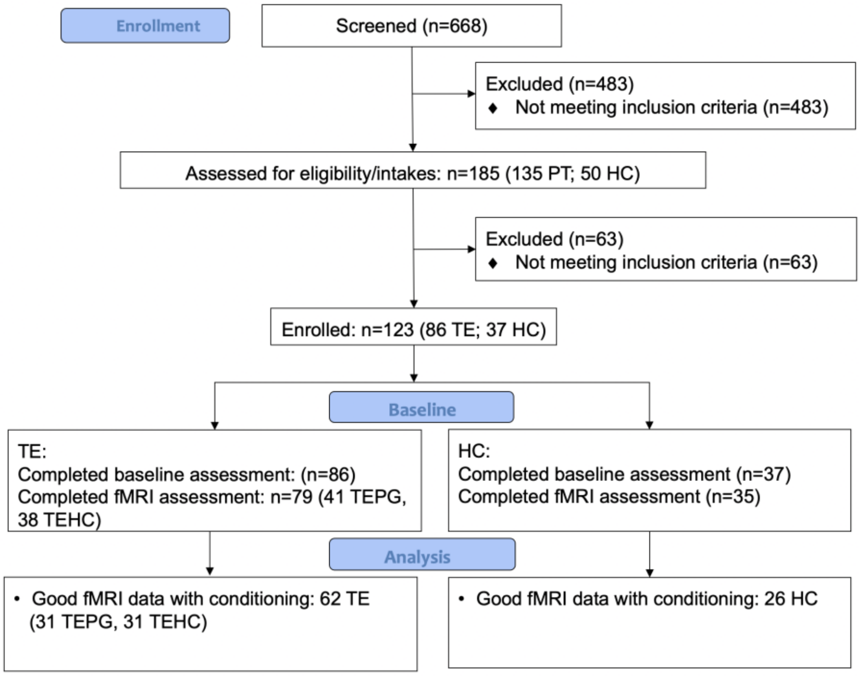

Figure S5: Flowchart of study protocol. 668 participants were screened for this protocol, among these, 483 participants did not meet inclusion criteria. 185 participants were assessed for eligibility and 123 participants were enrolled in this study. 114 participants (79 TEs and 35 HCs) completed both baseline clinical assessment and the fMRI assessment. Of the 79 TE participants, 41 had psychopathology at the level of a psychiatric diagnosis (TEPG), and 38 were trauma-exposed healthy controls with no diagnosis (TEHC). From the 114 sample, four participants were excluded due to being late for scan and not able to finish the SG task (two participants), or task failure of delivering shock (two participants). Twenty-two additional participants (15 TE and 7 HC) were excluded with greater risk rating to vCS-, compared to CS+ during acquisition phase in the fMRI task, or greater risk rating to vCS-, compared to red cross in post questionnaires. Consequently, the final neural imaging analysis included 88 participants: 62 TE and 26 HC.

Formatted: Space Before: Auto, After: Auto, Line spacing: single

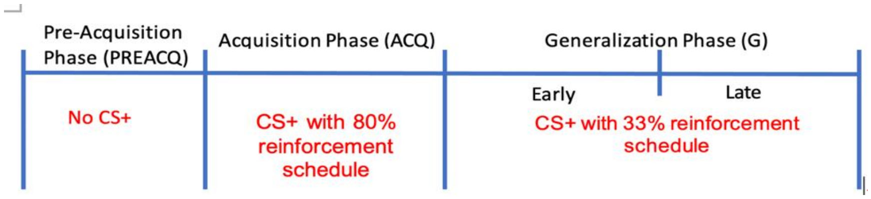

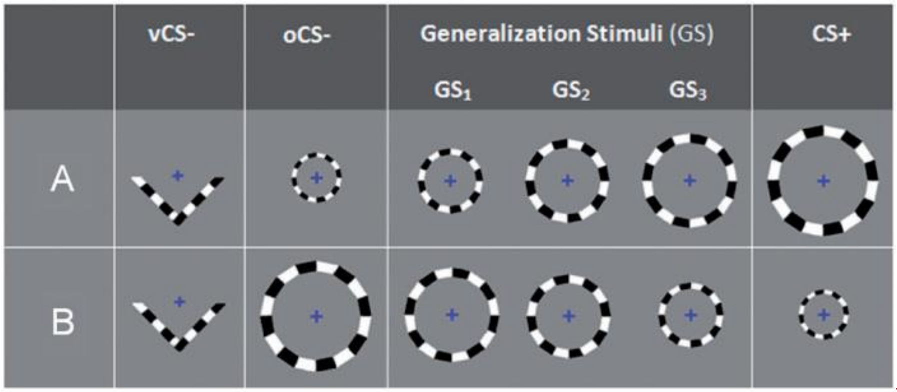

Formatted: Don't adjust space between Latin and Asian text, Don't adjust space between Asian text and numbers

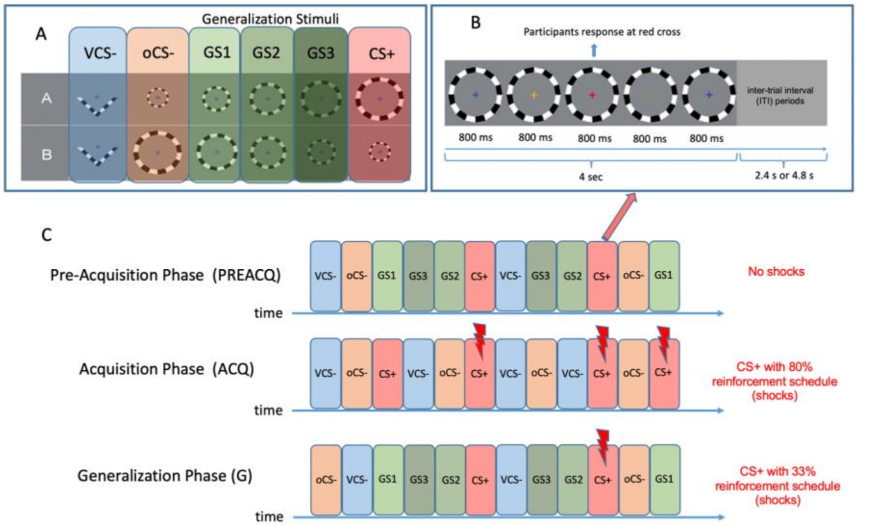

Figure S6:
A. This task consists of six types of stimuli including conditioned stimuli (CS+: conditioned danger cue; oCS-: o shape conditioned safe cue; vCS-: v shape conditioned safe cue) and three generalization stimuli (GSs including GS1, GS2, GS3). Half of the participants were assigned to counterbalancing group A and half to B. For both counterbalancing groups A and B, GS3 consisted of the ring closest in size to CS., with GS2 and GS1 further decreasing in similarity to CS+.
B. An example event sequence for CS+. There were a string of colored crosshairs (blue, yellow, red, green, and purple) presented serially for a duration of 800 ms each, in a quasi-random order in the center of the viewing screen during 4 s presentation of each stimulus. The inter-trial interval (ITI) periods last 2.4 s (three crosshairs) or 4.8 s (four crosshairs). Participants were instructed to continuously monitor the stream of colored crosshairs and rate their perceived level of risk for shock as quickly as possible following each red cross using a three-button fiber optic response pad, where 0='no risk', 1='moderate risk' and 2='high risk'.
C. Tthe generalization paradigm timeline included three phases-; (i) pre-acquisition-consisting in the absence of any shock US; (ii) acquisition-including with shock at an 80% reinforcement schedule; and (iii) generalization-including an early generalization (EG) stage and late generalization (LG) stage with shock US at a 33% reinforcement schedule to prevent extinction of the conditioned response. Conditioning and generalization stimuli for counterbalancing groups A and B. Half of the participants were assigned to counterbalancing group A and half to B. For both counterbalancing groups A and B, GS3 consisted of the ring closest in size to CS., with GS2 and GS1 further decreasing in similarity to CS+. Ring diameters in centimeters (and visual angles) from smallest to largest were: 6.63 (0.938), 8.02(1.128), 9.38 (1.318), 10.98 (1.548) and 12.46 (1.758).
vCS-=v-shaped conditioned safety cue; oCS-=ring-shaped conditioned safety cue; GS1, GS2 and GS3.three classes of generalization stimuli; CS+=conditioned danger cue.

Table S1: Summary of Independent Component Regions of Interest

	Area	Brodmann Area	volume (cc)	peak MNI (x, y, z)
SN	Posterior Insula	13, 22, 40	2.8/3.6	(-44, -16, 4)/(46, -14, 2)
	rACC	24, 32	0.3/0.4	(-4, 32, -8)/(2, 32, -10)
LECN	Inferior Parietal Lobule	39, 40	3.6/0.6	(-54, -62, 40)/(58, -56, 40)
	Middle Frontal Gyrus	8, 9	1.3/0.0	(-46, 22, 44)/(0, 0, 0)
RECN	Inferior Parietal Lobule	7, 39, 40	0.1/6.2	(-50, -62, 44)/(50, -54, 52)
	Superior Parietal Lobule	7	0.0/0.6	(0, 0, 0)/(42, -64, 50)
	Superior Frontal Gyrus	9, 10	0.0/0.3	(0, 0, 0)/(34, 62, -4)
a-DMN	Medial Frontal Gyrus	6, 9, 10	2.2/1.6	(-2, 64, 24)/(6, 62, 24)
	Superior Frontal Gyrus	9, 10	3.8/4.0	(-4, 62, 30)/(10, 64, 30)
	Middle Frontal Gyrus	*	0.0/0.3	(0, 0, 0)/(22, 62, 24)
p-DMN	Posterior Cingulate	23, 29, 30, 31	1.9/1.5	(0, -52, 20)/(4, -44, 20)
	Cingulate Gyrus	23, 31	2.3/1.3	(0, -40, 26)/(4, -40, 26)
	Precuneus	7, 31	3.6/3.1	(0, -68, 34)/(4, -70, 38)

Location names, Montreal Neurological Institute coordinates, extant voxels, and Brodmann areas are presented for each of the independent components with functional network connections. Display threshold at z>1.

Table S2. Analysis and results for behavioral markers (risk rating)

Analysis	Effects	Pre-ACQ	ACQ	SG1	SG2
ANOVA 2 groups (trauma)	Group by Stimulus-type	NS	NS	NS	NS
	Stimulus-type	NS	(Wilks' Lambda=0.74, F=14.58, p<0.001)	(Wilks' Lambda=0.68, F=7.24, p<0.001)	(Wilks' Lambda=0.50, F=14.28, p<0.001)
	Group	NS	NS	NS	NS
ANOVA 3 groups (resilience)	Group by Stimuli	NS	NS	NS	(Wilks' Lambda=0.77, F=1.91, p=0.049)
	Stimulus-type	NS	(Wilks' Lambda=0.69, F=18.38, p<0.001)	(Wilks' Lambda=0.64, F=8.57, p<0.001)	(Wilks' Lambda=0.45, F=16.78, p<0.001)
	Group	NS	NS	NS	NS
t-test HC	vCS- vs oCS-	p=0.144	p=0.012	p=0.478	p=0.235
	vCS- vs GS1	p=0.486	-	p=0.401	p=0.106
	vCS- vs GS2	p=0.267	-	p=0.052	p=0.002
	vCS- vs GS3	p=0.117	-	p=0.011	p=0.007
	vCS- vs CS+	p=0.094	p=0.009	p=0.002	p=0.000
t-test TE	vCS- vs oCS-	p=0.413	p=0.011	p=0.001	p=0.000
	vCS- vs GS1	p=0.938	-	p=0.000	p=0.000
	vCS- vs GS2	p=0.286	-	p=0.000	p=0.000
	vCS- vs GS3	p=0.178	-	p=0.000	p=0.000
	vCS- vs CS+	p=0.385	p=0.000	p=0.000	p=0.000
t-test TEHC	vCS- vs oCS-	p=0.262	p=0.027	p=0.057	p=0.034
	vCS- vs GS1	p=0.035	-	p=0.002	p=0.039
	vCS- vs GS2	p=0.348	-	p=0.000	p=0.000
	vCS- vs GS3	p=0.078	-	p=0.000	p=0.000
	vCS- vs CS+	p=0.882	p=0.000	p=0.000	p=0.000
t-test TEPG	vCS- vs oCS-	p=0.72	p=0.175	p=0.004	p=0.001
	vCS- vs GS1	p=0.232	-	p=0.006	p=0.000
	vCS- vs GS2	p=0.434	-	p=0.000	p=0.000
	vCS- vs GS3	p=0.783	-	p=0.000	p=0.000
	vCS- vs CS+	p=0.394	p=0.000	p=0.000	p=0.000
