# Supplementary material for: Sequential fear generalization and network connectivity in trauma exposed humans with and without psychopathology
Source: Commun Biol. 2022 Nov 21;5:1275. doi: 10.1038/s42003-022-04228-5 (PMC9681725; doi:10.1038/s42003-022-04228-5)
Supplement: Supplementary file 3 — Description of Additional Supplementary Files [file 42003_2022_4228_MOESM3_ESM.pdf]

Description of Additional Supplementary Files

File name: Supplementary Data 1

Description: Data used for visualizations in Figure, 1, 2, 3
