# Supplementary material for: Sequential fear generalization and network connectivity in trauma exposed humans with and without psychopathology
Source: Commun Biol. 2022 Nov 21;5:1275. doi: 10.1038/s42003-022-04228-5 (PMC9681725; doi:10.1038/s42003-022-04228-5)
Supplement: Supplementary file 5 — Reporting Summary [file 42003_2022_4228_MOESM5_ESM.pdf]

nature portfolio

Corresponding author(s): Yuval Neria

Last updated by author(s): Sep 14, 2022

Reporting Summary

Nature Portfolio wishes to improve the reproducibility of the work that we publish. This form provides structure for consistency and transparency in reporting. For further information on Nature Portfolio policies, see our Editorial Policies and the Editorial Policy Checklist.

Statistics

For all statistical analyses, confirm that the following items are present in the figure legend, table legend, main text, or Methods section.

n/a	Confirmed	
☐	☒	The exact sample size (*n*) for each experimental group/condition, given as a discrete number and unit of measurement
☐	☒	A statement on whether measurements were taken from distinct samples or whether the same sample was measured repeatedly
☐	☒	The statistical test(s) used AND whether they are one- or two-sided<br>*Only common tests should be described solely by name; describe more complex techniques in the Methods section.*
☐	☒	A description of all covariates tested
☐	☒	A description of any assumptions or corrections, such as tests of normality and adjustment for multiple comparisons
☐	☒	A full description of the statistical parameters including central tendency (e.g. means) or other basic estimates (e.g. regression coefficient) AND variation (e.g. standard deviation) or associated estimates of uncertainty (e.g. confidence intervals)
☐	☒	For null hypothesis testing, the test statistic (e.g. *F*, *t*, *r*) with confidence intervals, effect sizes, degrees of freedom and *P* value noted<br>*Give P values as exact values whenever suitable.*
☒	☐	For Bayesian analysis, information on the choice of priors and Markov chain Monte Carlo settings
☒	☐	For hierarchical and complex designs, identification of the appropriate level for tests and full reporting of outcomes
☐	☐	Estimates of effect sizes (e.g. Cohen's *d*, Pearson's *r*), indicating how they were calculated

Our web collection on statistics for biologists contains articles on many of the points above.

Software and code

Policy information about availability of computer code

Data collection	No software was used for data collection
Data analysis	Matlab version 2020b was used for imaging analysis

For manuscripts utilizing custom algorithms or software that are central to the research but not yet described in published literature, software must be made available to editors and reviewers. We strongly encourage code deposition in a community repository (e.g. GitHub). See the Nature Portfolio guidelines for submitting code & software for further information.

Data

Policy information about availability of data

All manuscripts must include a data availability statement. This statement should provide the following information, where applicable:

- Accession codes, unique identifiers, or web links for publicly available datasets
- A description of any restrictions on data availability
- For clinical datasets or third party data, please ensure that the statement adheres to our policy

The datasets generated during the current study are available from the corresponding author on reasonable request.

Human research participants

Policy information about studies involving human research participants and Sex and Gender in Research.

Reporting on sex and gender	Findings apply to male and female sex. Sex was determined based on self-reporting. Overall numbers are provided in Table 1. Effects of covariate corresponding to different scanners, age and sex was used in all analysis as a covariate of no interest.
Population characteristics	See below.
Recruitment	Participants were recruited by local advertisements, websites, and word-of-mouth referrals and evaluated at the New York State Psychiatric Institute (NYSPI).
Ethics oversight	The methods were performed in accordance with relevant guidelines and regulations and approved by NYSPI IRB.

Note that full information on the approval of the study protocol must also be provided in the manuscript.

Field-specific reporting

Please select the one below that is the best fit for your research. If you are not sure, read the appropriate sections before making your selection.

☐ Life sciences ☒ Behavioural & social sciences ☐ Ecological, evolutionary & environmental sciences

For a reference copy of the document with all sections, see nature.com/documents/nr-reporting-summary-flat.pdf

Behavioural & social sciences study design

All studies must disclose on these points even when the disclosure is negative.

Study description	Qualitative cross-sectional
Research sample	The research sample includes trauma exposed participants and healthy control recruited from New York City.
Sampling strategy	The research sample includes trauma exposed participants and healthy control recruited from New York City. To ensure substantial range of psychopathology in the TE sample, and consistent with a large body of work suggesting dose-response relationships between trauma exposure severity and psychopathology (29), we methodically characterized type, severity, number and timing of trauma exposures, and included both childhood and adulthood trauma exposure. Index trauma exposure met DSM-5 Criterion A of a traumatic event.
Data collection	An independent MD or PhD/PsyD clinical evaluator administered the Structured Clinical Interview for DSM-5 Disorders (SCID-5)(30) and the Clinician-Administered PTSD Scale-5 (CAPS-5) (31, 32). Depressive symptoms were assessed using the Hamilton Depression Scale (HAM-D, 17 item) (34), a reliable clinician-rated measure of depressive severity. Social anxiety symptoms were assessed using the Liebowitz Social Anxiety Scale (LSAS). Panic disorder symptoms were assessed using the Panic Disorder Severity Scale (PDSS)(36), a self-report measure for panic severity. Self-rated instruments also included the Generalized Anxiety Disorder assessment (GAD-7) (37), and the SF-36 (38), a 36 item measure of generic health status, designed for use in clinical practice and research.
Timing	12/21/15-8/15/19
Data exclusions	From the original 114 sample, four participants were excluded due to being late for scan and not able to finish the SG task (two participants), or task failure of delivering shock (two participants). Twenty-two additional participants (15 TE and 7 HC) were excluded with greater risk rating to vCS-, compared to CS+ during acquisition phase in the fMRI task, or greater rating to vCS-, compared to red cross in post questionnaires. No participant was excluded from further analysis because of movement exceeding ±1 mm. Consequently, the final neural imaging analysis included 88 participants: 62 TE and 26 HC.
Non-participation	No participants dropped out after enrolled.
Randomization	NA

Reporting for specific materials, systems and methods

We require information from authors about some types of materials, experimental systems and methods used in many studies. Here, indicate whether each material, system or method listed is relevant to your study. If you are not sure if a list item applies to your research, read the appropriate section before selecting a response.

Materials & experimental systems

n/a	Involved in the study
☒	☐ Antibodies
☒	☐ Eukaryotic cell lines
☒	☐ Palaeontology and archaeology
☒	☐ Animals and other organisms
☒	☐ Clinical data
☒	☐ Dual use research of concern

Methods

n/a	Involved in the study
☒	☐ ChIP-seq
☒	☐ Flow cytometry
☐	☒ MRI-based neuroimaging

Magnetic resonance imaging

Experimental design

Design type

Event-related design

Design specifications

The generalization paradigm included three phases (Figure S6): (i) pre-acquisition-consisting of 20 trials of each stimulus type (CS+, GS1, GS2, GS3, oCS- and vCS-), all presented in the absence of any shock US; (ii) acquisition-including 15 CS+, 15 oCS-, and 15 vCS-, with 12 of 15 CS+ co-terminating with shock (80% reinforcement schedule; (14) and (iii) generalization-including an early generalization (EG) stage and late generalization (LG) stage, each comprised of 10 trials of each stimulus type (unreinforced CS+, GS1, GS2, GS3, oCS-, vCS-) and an additional 5 CS+ co-terminating with shock (33% reinforcement schedule) to prevent extinction of the conditioned response while leaving 10 unreinforced CS+ to index responses uninfluenced by the shock US. Trials of all three phases were arranged in quasi-random order such that no more than two stimulus-type of the same class occurred consecutively. An additional constraint for the generalization phase was the arrangement of trials into six blocks of 13 trials each (i.e., two unreinforced CS+, one reinforced CS+, two oCS-, two vCS-, two GS1, two GS2, two GS3) to ensure an even distribution of trial types throughout runs.

Behavioral performance measures

Response time was recorded during the task. mean and standard deviation were used to establish that the subjects were performing the task as expected.

Acquisition

Imaging type(s)

functional MRI

Field strength

3T

Sequence & imaging parameters

T1-weighted 3D BRAVO sequence was acquired using the following parameter: T1=450 mm, Flip angle =12°, field of view =25.6 cm, 256×256 matrix, slice thickness =1 mm. Whole brain T2*-weighted echo-planar images (EPIs) depicting the blood-oxygen-level-dependent (BOLD) were acquired for each participant with TR=1.3 sec, TE=28 msec, FA =60°, FOV =19.2 cm, number of slices=27, slice thickness=4 mm.

Area of acquisition

whole brain scan was used.

Diffusion MRI ☐ Used ☒ Not used

Preprocessing

Preprocessing software

All fMRI images were preprocessed using MATLAB version R2020b (The MathWorks, Inc., Natick, Massachusetts) and statistical parametric mapping software (SPM12; Welcome Trust Centre for Neuroimaging, UCL, London, United Kingdom).

Normalization

Each functional image was then spatially normalized to the standard T1 template included in SPM12; functional images were then re-sliced to 2×2×2 mm voxels, according to the resulting spatial realignment and normalization parameters.

Normalization template

MNI152

Noise and artifact removal

Covariates corresponding to head motion (6 realignment parameters and their derivatives), outliers (one covariate per outlier consisting of 0s everywhere and a 1 for the outlier time point), and the BOLD time series from the participant-specific white matter and CSF masks were used in the GLM and connectivity analysis as predictors of no interest, and were removed from the BOLD functional time series using linear regression.

Volume censoring

Outlier detection was carried out using artifact removal tools (ART). The principal component-based noise-correction method, “CompCor,” implemented in this toolbox, was used for additional control of physiological noise and head motion effects. Outlier volumes in each participant were identified as having large spiking artifacts (i.e., volumes >3 standard deviations from the mean image intensity), or large motion (i.e., 0.5 mm for scan-to-scan head-motion composite changes in the x, y, or z direction).

Statistical modeling & inference

Model type and settings	multivariate data analysis (independent component analysis) was used in this study.
Effect(s) tested	To examine the generalization phases over time (EG, LG), for both behavior and neural markers, we assessed changes (delta) in behavioral and neural markers over the two stages (EG-LG) for trauma exposure by comparing TE and HC using a group (TE and HC) by stimulus-type (vCS-, oCS-, GS1, GS2, GS3 and unreinforced CS+) repeated measures ANOVAs.

Specify type of analysis: ☒ Whole brain ☐ ROI-based ☐ Both

Statistic type for inference (See Eklund et al. 2016)	Voxel-wise analysis
Correction	Bonferroni correction was used to compare the averaged network identified by ICA.

Models & analysis

n/a	Involved in the study
☐	☒ Functional and/or effective connectivity
☒	☐ Graph analysis
☒	☐ Multivariate modeling or predictive analysis

Functional and/or effective connectivity	Functional connectivity was measured using independent component analysis.
